# Comprehensive analysis of aberrant alternative splicing related to carcinogenesis and prognosis of papillary thyroid cancer

**DOI:** 10.18632/aging.203608

**Published:** 2021-10-08

**Authors:** Xiaobo Zheng, Li Feng, Yunfan Yin, Chune Yu, Xiujing He, Jiao Zhu, Ming Zhang, Jing Yu, Mingqing Xu

**Affiliations:** 1 Department of Liver Surgery, West China Hospital, Sichuan University, Chengdu 610041, Sichuan, China; 2 Department of General Surgery, Hospital of Chengdu University of Traditional Chinese Medicine, Chengdu 610072, Sichuan, China; 3 Department of Otolaryngology Head and Neck Surgery, General Hospital of Western Theater Command, Chengdu 610083, Sichuan, China; 4 Laboratory of Tumor Targeted and Immune Therapy, Clinical Research Center for Breast, State Key Laboratory of Biotherapy, West China Hospital, Sichuan University, Chengdu 610041, Sichuan, China; 5 Hearing Center/Hearing and Speech Laboratory, Department of Otorhinolaryngology Head and Neck Surgery, West China Hospital, Sichuan University, Chengdu 610041, Sichuan, China; 6 Department of General Surgery, Mianzhu Hospital of West China Hospital, Sichuan University, Mianzhu 618200, Sichuan, China; 7 Department of Hepatopancreatobiliary Surgery, Meishan Hospital of West China Hospital, Sichuan University, Meishan City People’s Hospital, Meishan 610020, Sichuan, China

**Keywords:** papillary thyroid cancer, alternative splicing, carcinogenesis, prognosis, The Cancer Genome Atlas

## Abstract

As a key mechanism, alternative splicing (AS) plays a role in the cancer initiation and development. However, in papillary thyroid cancer (PTC), data for the comprehensive AS event profile and its clinical implications are lacking. Herein, a genome-wide AS event profiling using RNA-Seq data and its correlation with matched clinical information was performed using a 389 PTC patient cohort from the project of The Cancer Genome Atlas (TCGA). We identified 1,925 cancer-associated AS events (CASEs) by comparing paired tumors and neighboring healthy tissues. Parent genes with CASEs remarkably enriched in the pathways were linked with carcinogenesis, such as P53, KRAS, IL6-JAK-STAT3, apoptosis, and MYC signaling. The regulatory networks of AS implied an obvious correlation between the expression of splicing factor and CASE. We identified eight CASEs as predictors for overall survival (OS) and disease-free survival (DFS). The established risk score model based on DFS-associated CASEs successfully predicted the prognosis of PTC patients. From the unsupervised clustering analysis results, it is found that different clusters based on AS correlated with prognosis, molecular features, and immune characteristics. Taken together, the comprehensive genome-wide AS landscape analysis in PTC showed new AS events linked with tumorigenesis and prognosis, which provide new insights for clinical monitoring and therapy for PTC.

## INTRODUCTION

Thyroid cancer (TC) is the most prevailing endocrine cancer globally; as its main subtype, papillary thyroid cancer (PTC) occupies over 90% of all TCs [1, 2]. PTC morbidity has rapidly increased over the past decades, which has raised substantial concern [3]. While PTC is usually indolent, some patients still develop recurrence and metastasis even after radical surgical resection, resulting in cancer-related death [4, 5]. Early recognition of high-risk patients will benefit precise individual therapy and avoid overtreatment in low-risk patients. Therefore, the biomarkers must be determined to recognize patients with PTC for effective early prevention and clinical treatment.

The rapid development of next-generation sequencing technologies contributed to improvement in cancer genomics research [6]. Using a multi-omics approach in recent years, the molecular characteristics of PTC have been extensively studied in the aspects of genomics, transcripts, and proteins [7–11]. Despite this, comprehensive analysis of the variations in alternative splicing (AS) of PTC is rarely performed. Being a common physiological process, AS can splice a pre-mRNA in distinct models to generate structurally and functionally different transcripts and protein variants [12, 13]. Through these extensively applied mechanisms, proteome diversity has been shown to regulate various physiological processes [14, 15]. Considering the profound effect of AS on biological processes, disruptions in AS could contribute to the development of human diseases such as cancer [16–18]. For instance, when some precursor mRNAs which are functionally linked encounter AS dysregulation, the whole protein structure could be altered, which facilitates tumor carcinogenesis [19]. Moreover, incremental evidence has revealed that the dysregulated splicing isoform expression or the imbalance in the correct isoform expression is a common cancer phenotype [20]. Hence, cancer-specific AS can be a potential diagnostic and prognostic predictor and a new target for precise therapy.

Currently, profiling of the AS landscape in different types of cancer is available using RNA sequencing data and matched clinical information from the project of The Cancer Genome Atlas (TCGA). Recently, increasing evidence showed that AS plays an active part in tumor carcinogenesis in various types of cancer [21–25]. Hence, it is feasible to explore the clinical significance of AS events related to PTC using relatively massive sequencing data. Teng et al. profiled the transcriptomic signature in PTC which were linked with carcinogenesis and aggressiveness [26]. Differential AS events and their potential regulatory mechanisms were also identified, which contributed to a transcriptome-wide understanding of PTC [26]. However, the correlation between dysregulation of splicing patterns and clinical prognosis in PTC is still poorly understood.

To explore the landscape of abnormal AS and its clinical significance in PTC, the relationship between integrated AS profiles and complete TCGA clinical information was examined. In this study, AS events which were genome-wide were comprehensively profiled and the cancer-associated AS events (CASEs) in PTC were recognized. The underlying biological functions and potential regulatory mechanisms of these CASEs were further investigated. To this end, a regulatory network was constructed between splicing factors (SFs) and CASEs. Survival-associated CASEs were identified and a risk model was thus established for predicting PTC patients’ prognosis. Moreover, we identified the distinct clusters of PTC-associated AS events and explored the relationship between clusters based on AS and clinical pathological variables and tumor microenvironment (TME).

## RESULTS

### Summary of AS event profiling in PTC cohort from the TCGA database

395 PTC patients were recognized in the TCGA database. Among them, 389 patients, for whom RNA-seq data and complete clinical information were securable, were enrolled in the next analysis. Meanwhile, 6 patients without histological diagnosis, complete clinical information, or corresponding RNA-seq data were excluded. Table 1 presents the baseline demographic features and histopathological characteristics of the 389 enrolled patients. 440 samples (389 tumor samples and 51 matched adjacent normal tissues) were included. In the follow-up months ranging from 0 to 178.1, the median was 31.3 months; 38 (10.1%) patients experienced recurrence/progression and 15 (3.8%) patients deceased. The 3-year OS rate was 96.8% and 3-year DFS rate was 88.7%.

**Table 1:** Clinical characteristics of enrolled papillary thyroid cancer patients.

	**NO.**	**OS**		**DFS**	
		**Hazard rations (95%CI)**	**P value**	**Hazard rations (95%CI)**	**P value**
Age					
≥60	92	55.284 (7.253–421.414)	0	2.215(1.132–4.336)	0.02*
<60	297				
Gender					
Male	107	2.018(0.715–5.698)	0.185	1.285(0.648–2.547)	0.473
Female	282				
TNM stage					
Stage I-II	249	9.32(2.621–33.139)	0.001*	2.459(1.298–4.658)	0.006*
Stage III-IV	139				
NA	1				
Tumor stage					
T1-2	231	3.607(1.147–11.344)	0.028*	2.082(1.093–3.967)	0.026*
T3-4	156				
NA	2				
Lymph node metastasis					
N0	159	1.113(0.363–3.41)	0.852	1.824(0.897–3.708)	0.097
N1	204				
NA	26				
Distant metastasis					
M0	245	8.771(1.846–41.686)	0.006*	3.585(0.472–27.246)	0.217
M1	3				
NA	141				
Tumor status					
Tumor free	309	33.189(6.866–160.444)	0	8.287(4.32–15.895)	0
With tumor	44				
NA	36				

NA, not available; CI, confidence interval; *, P<0.05.

**Figure 1:**
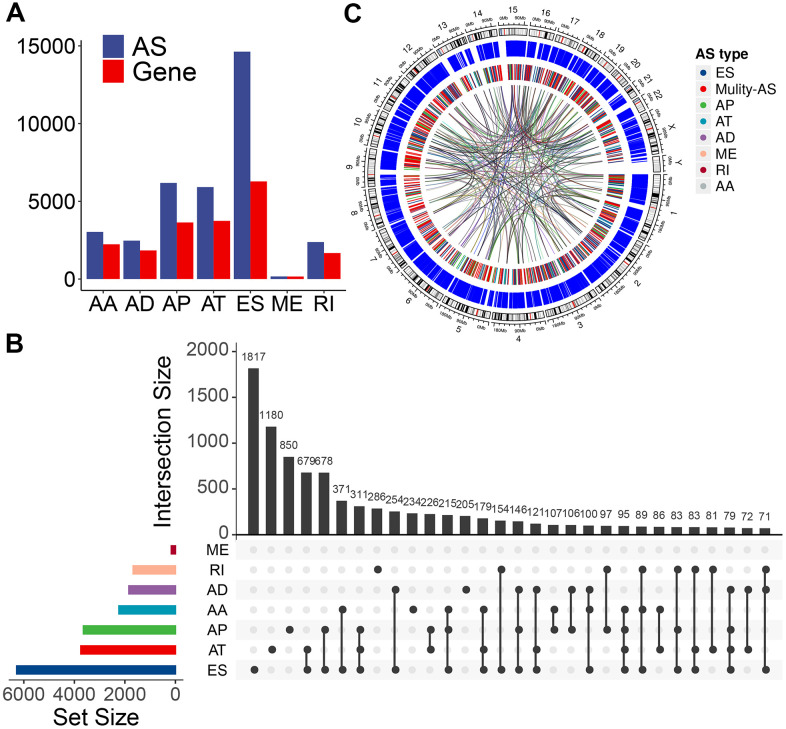
**Overview of AS event profiling in the TCGA PTC cohort.** (**A**) Number of each AS event type and their parent genes in PTC patients. Blue bars represent the AS events, while red bars represent their parent genes. (**B**) Interactive sets among seven types of AS events (n = 34,773) shown in an UpSet plot. (**C**) Circos plot shows the details of each AS event and their parent genes in the chromosome. The outer circle represents the chromosome ideogram. The intermediate circle represents the genes with filtered AS events. The inner circle shows the genes with differentially expressed AS events between tumor and normal tissues. The ribbons represent the potential interaction between AS events and their parent genes. ES, exon skipping; AP, alternate promoter; AT, alternate terminator; AD, alternate donor; AA, alternate acceptor site; ME, mutually exclusive exons; RI, retained intron. Mulity-AS, gene contains multiple types of AS.

We used these enrolled patients’ RNA-Seq data to set up integrated profiling of AS events. After downloading the percent spliced index (PSI) values for AS events in PTC from the databank of TCGA SpliceSeq [27] (http://bioinformatics.mdanderson.org/TCGASpliceSeq/), we initially identified 34,773 AS events from 10,219 genes after applying the most stringent filters (percent of samples with ≥75 PSI values; mean PSI value: ≥0.05). These AS events were divided into seven splicing types: 14,630 exon skipping (ES) events from 6,281 genes, 6,181 alternate promoters (AP) from 3,641 genes, 5,916 alternate terminators (AT) from 3,744 genes, 2,469 alternate donor (AD) sites from 1,842 genes, 3,032 alternate acceptor (AA) sites from 2,236 genes, 166 mutually exclusive exons (ME) from 162 genes, and 2,379 retained introns (RI) from 1,672 genes (Figure 1A). In these splicing types, the ES occurrence was the most frequent (42.1%), followed by AP (17.8%), AT (17.0 %), and AA (8.7%) (Figure 1A). Considering a single gene may have multiple splicing modes, we quantitatively visualized the interactive sets of each AS type by generating Upset plots. Most AS events were from one gene; meanwhile, an individual gene may have four distinct splicing types, and most of the genes showed multiple AS events (Figure 1B), implying the biggest possible process for the transcriptome diversity enrichment. Moreover, we created Circos plots to show the details of PTC AS event profiling (Figure 1C).

### Identification of CASEs in PTC

**Figure 2:**
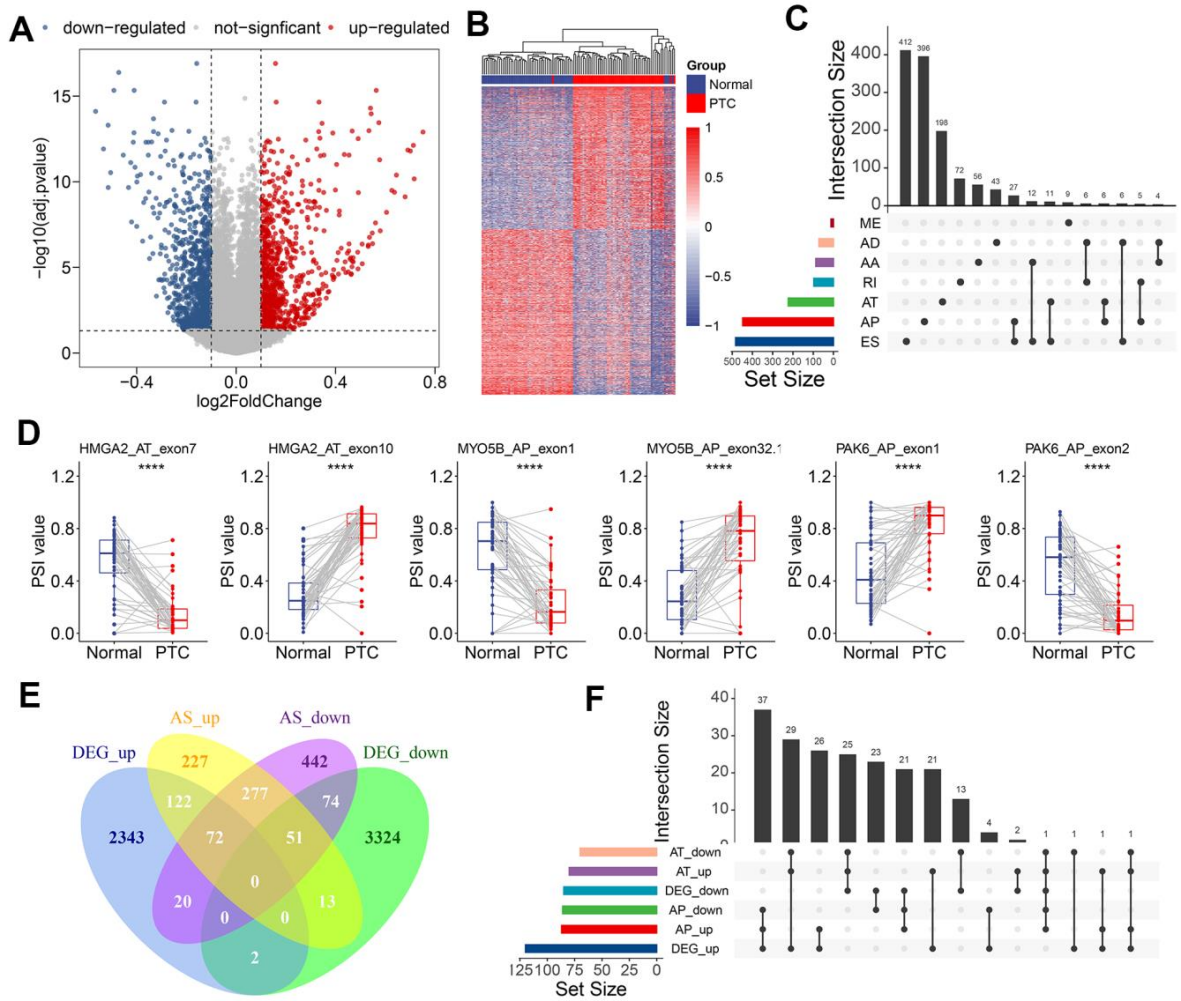
**Identification of CASEs in PTC.** (**A**) Volcano plot of CASEs identified in PTC (log_2_^FC^ > 0.1, adjusted P < 0.05). (**B**) Heatmap of the CASEs between matched tumor and adjacent normal tissues of 50 PTC patients. (**C**) Interactive sets among seven AS types of CASEs (n = 1,925) shown in an UpSet plot. (**D**) The representative CASEs, derived from the same parent gene and exhibited the opposite preference between tumor and adjacent normal tissues, were shown. Student’s t-test was used. (**E**) Venn diagram of CASEs and DEGs. (**F**) Interactive sets of AP/AT events and DEGs shown in UpSet plot. ES, exon skipping; AP, alternate promoter; AT, alternate terminator; AD, alternate donor; AA, alternate acceptor site; ME, mutually exclusive exons; RI, retained intron.

To identify the CASEs during PTC carcinogenesis, we compared each AS event’s PSI values between 50 paired tumors and adjacent normal tissues. 1,925 CASEs from 1,298 genes were screened with the threshold set to |log_2_^FC^| >0.1 and with a modified P-value of <0.05 (BH correction) (Figure 2A). Using CASE-based unsupervised hierarchical clustering, the tumor samples and healthy tissue samples were obviously divided into two distinct groups, suggesting that CASEs accurately distinguished the tumors from the normal tissues in PTC (Figure 2B). Among these CASEs, there were 687 APs, 585 ESs, 366 ATs, 102 RIs, 97 AAs, 76 ADs, and 12 MEs (Figure 2C). Although a significantly larger number of ES events were detected in all AS events, similar proportions of ES and AP events were recognized as CASEs, followed by AT CASEs (Figure 2C). These inconsistent distributions between CASE patterns and all AS events indicated that each AS type may play different parts in development of cancer. Accordingly, some genes (like HMGA2, MYO5B, and PAK6) had the opposite roles of AS events from the same parental gene in tumor and healthy tissues (Figure 2D). To validate the recurrence of identified CASE events in another independent cohort, we analyzed the CASEs in head and neck squamous cell carcinoma (HNSC) and colon adenocarcinoma (COAD) which have alternative splicing information from TCGA SpliceSeq datasets. Interestingly, among the 1,925 CASEs in PTC, 97 were differentially expressed in HNSC and COAD (Supplementary Figure 1A). For example, AT of exon 5.2 in CXCL12 was remarkably upregulated, while AT of exon 3.3 in CXCL12 was downregulated in PTC, HNSC and COAD (Supplementary Figure 1B). These findings suggested that AS events we identified were shared in tumorigenesis among various cancers.

Abnormal AS events can alter its parental RNA expression straightway, and AT/AP events were more preferred for this phenomenon. Consistent with this notion, 57.9% of CASEs in the corresponding differentially expressed gene (DEG) in PTC were AT (26.4%) and AP (31.5%). To illustrate the potential effect of CASEs on DEGs, we analyzed the relationship between CASEs and DEGs. We observed an overlap between CASEs and DEGs by intersection analysis (Figure 2E). The intersection analysis showed that AT/AP events had a relationship with parental gene dysregulation (Figure 2F), implying that CASEs may promote tumor development by aberrantly regulating the gene expression. Collectively, these evidences suggested that PTC-related AS events may play a functional role in PTC carcinogenesis.

### Tumor carcinogenesis-related signature enriched by CASEs in PTC

**Figure 3:**
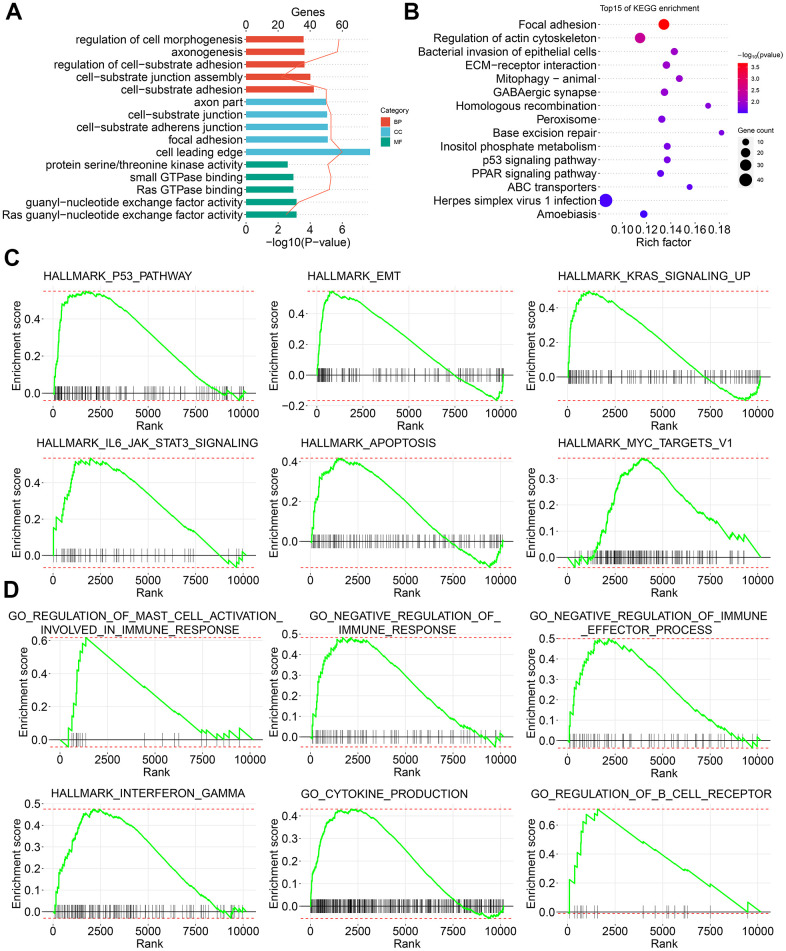
**Signature enrichment by CASEs in PTC.** (**A**) GO analysis of CASEs. (**B**) KEGG analysis of CASEs. (**C**, **D**) GSEA analysis of all AS events.

To reveal the molecular signatures of parent genes with CASEs, the enriched pathways of CASEs were analyzed using the biological function enrichment analysis. These parent genes with CASEs substantially enriched in GO pathways had a close relationship with the extracellular matrix, such as cell morphogenesis regulation, cell-substrate adhesion regulation, and focal adhesion (Figure 3A). Moreover, some molecular functions were highly enriched, such as Ras guanyl−nucleotide exchange factor activity, guanyl-nucleotide exchange factor activity, and Ras GTPase binding (Figure 3A). Additionally, several KEGG pathways linked with carcinogenesis were enriched, such as homologous recombination, base excision repair, inositol phosphate metabolism, p53 signaling pathway, and PPAR signaling pathway (Figure 3B). Consistent with these findings, the GSEA showed that AS events which were differentially expressed in PTC were remarkably enriched in the oncogenesis pathways, such as P53, epithelial mesenchymal transition, KRAS, IL6-JAK-STAT3, apoptosis, and MYC signaling (Figure 3C). Interestingly, immune-related pathways, such as interferon gamma response, negative regulation of immunoreaction, regulation of B cell receptor signaling pathway, positive mast cell activation regulation, chemokine production, and negative regulation of immune effector process, were also enriched (Figure 3D).

**Figure 4:**
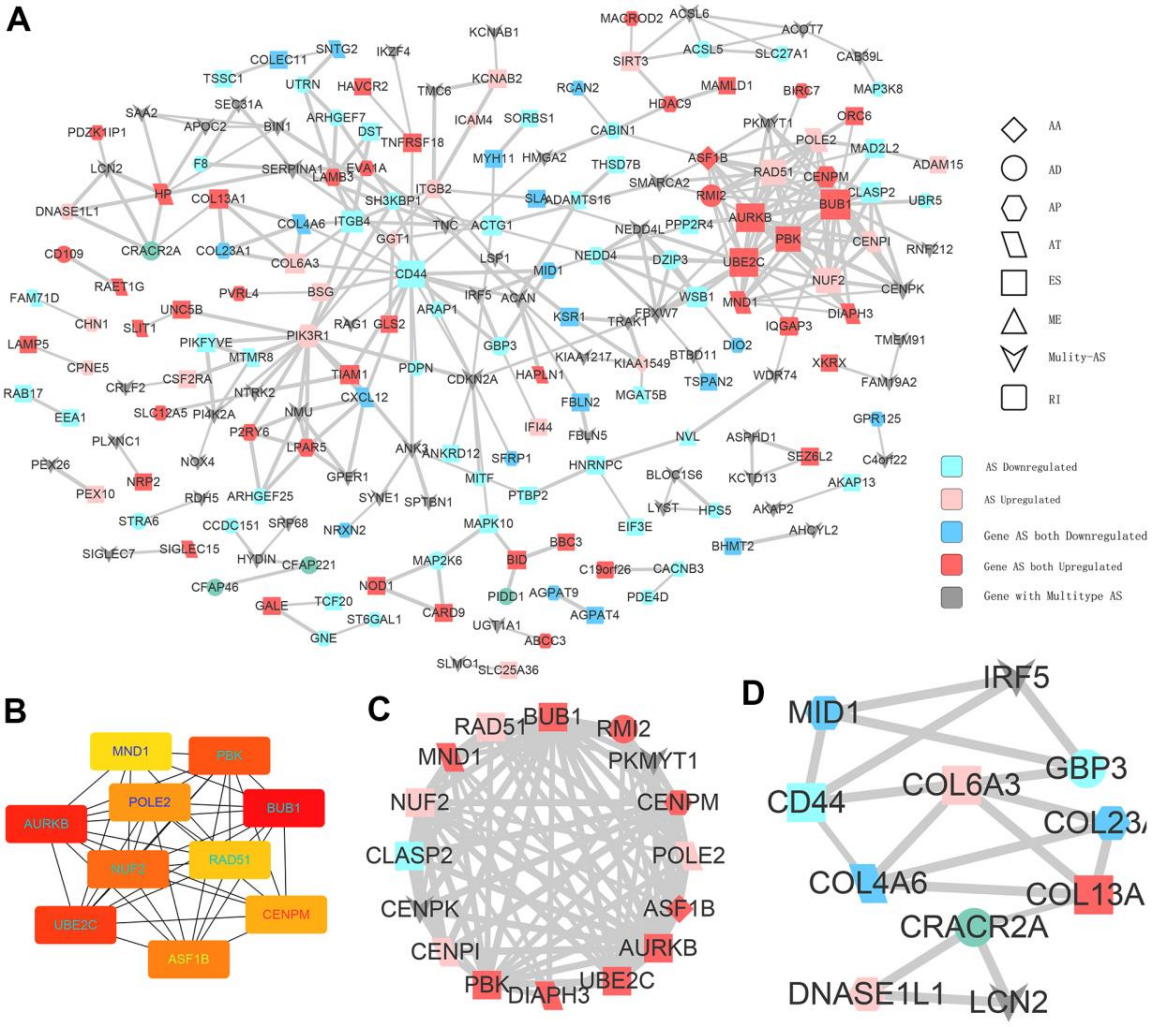
**Interaction analysis of CASEs.** (**A**) PPI network analysis of CASEs generated by Cytoscape. Nodes indicate parent genes with CASEs, while edges represent the potential interactions between the corresponding proteins. The shape, size, and color of nodes denote AS types, the value of log_2_^FC^, and change patterns, respectively. ES, exon skipping; AP, alternate promoter; AT, alternate terminator; AD, alternate donor; AA, alternate acceptor site; ME, mutually exclusive exons; RI, retained intron. (**B**) Hub genes ranked by MCC. (**C**) Module 1 was correlated with tumorigenesis. (**D**) Module 2 was correlated with extracellular matrix.

Given that AS could inevitably affect protein translation and produce various amino acid sequences, we investigated the PPI network of CASEs to determine the possible influence of AS events on the whole network (Figure 4A). Based on the PPI network, ubiquitin conjugating enzyme E2C (UBE2C) and aurora kinase B (AURKB) were identified as hub genes (Figure 4B). By analyzing the protein features, two PPI network modules were identified. In agreement with the outcomes in the enrichment analysis, CASEs in the first one were enriched in the carcinogenesis’s biological process (Figure 4C). The other module comprised CASEs enriched in the extracellular matrix (Figure 4D). Collectively, these results implied that the parental gene of CASEs plays significant roles in carcinogenesis and in the tumor immune microenvironment formation in PTC.

### Network of CASEs and SFs

**Figure 5:**
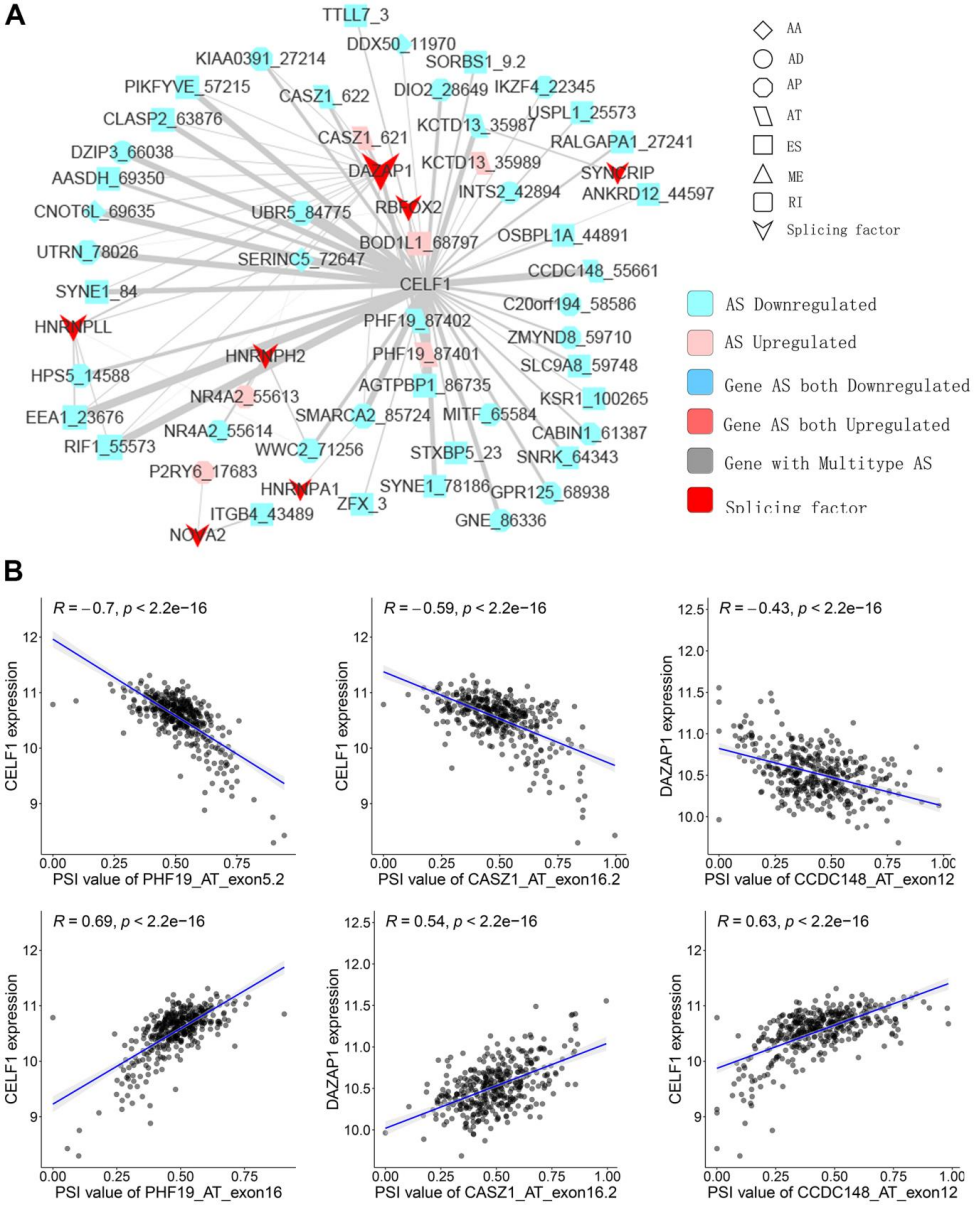
**Representative plots of regulatory splicing network in PTC.** (**A**) The correlation analysis between the expression levels of 71 SFs and the PSI values of CASEs. The shape, color, and size of node denote AS types, changes in the pattern (upregulated or downregulated), and the value of log_2_^FC^, respectively. The breadth of each line represents the extent of interaction strength. (**B**) Representative dot plots indicate the correlations between the expression of SFs and PSI values of CASEs.

SFs are the main AS event regulators by bonding to pre-mRNAs and influencing the exon selection or intron removal [28]. SFs can facilitate dysfunctional splicing patterns in tumors in comparison with healthy tissues, leading to the pro-tumorigenic isoform production [29, 30]. Hence, to illustrate how SFs regulate CASEs in PTC is of significance. The relationship between the expression of seventy-one experimentally validated SFs in a previous study [31] and CASEs’ PSI values was analyzed; a regulatory network on splicing among the significant associations was constructed. As shown in Figure 5A, 27 upregulated CASEs (pink dots) and 64 downregulated CASEs (blue dots) were associated with 27 SFs (red dots). Notably, the majority of SFs were remarkably linked with multiple AS events. Furthermore, a single AS event can be regulated by as many as nine different SFs; hence, transcripts can produce diverse splicing isoforms due to the competition of a few factors only. Some representative correlations of highly correlated SFs and CASEs are shown in Figure 5B. For instance, the CELF1 expression had a positive correlation with AT exon16 87402 of PHF19, but it was negatively correlated with AT exon5.2 87401 of PHF19.

### Survival-associated CASEs in PTC

As most CASEs do not play a functional role during carcinogenesis, we examined CASEs associated with PTC patient prognosis to identify CASEs involved in tumor biological processes. Before investigating the prognostic values of CASEs, the univariate survival analysis was performed to assess the correlation between the clinical pathological characteristics and PTC patients’ outcome in the TCGA cohort (Table 1). Table 1 shows that, age (hazard ratio [HR] = 2.215, 95% CI: 1.132–4.336; P = 0.02), American Joint Committee on Cancer TNM stage (HR = 2.46, 95% CI: 1.298–4.66; P = 0.006), tumor stage (HR = 2.08, 95% CI: 1.093–3.97; P = 0.026), and tumor status (HR = 16.566, 95% CI: 9.361–29.314; P < 0.001) were significantly associated with the DFS of PTC. Meanwhile, age (HR = 55.284, 95% CI: 7.253–421.414; P < 0.001), tumor stage (HR = 3.61, 95% CI: 1.147–11.34; P = 0.028), M stage (HR = 8.77, 95% CI: 1.846–41.69; P = 0.006), TNM stage (HR = 9.32, 95% CI: 2.621–33.14; P = 0.001), and tumor status (HR = 8.287, 95% CI: 4.32–15.895; P < 0.001) had a significant association with OS. These preliminary results indicated that the survival data of the TCGA PTC cohort were full of information and suitable for use in subsequent survival analysis.

**Figure 6:**
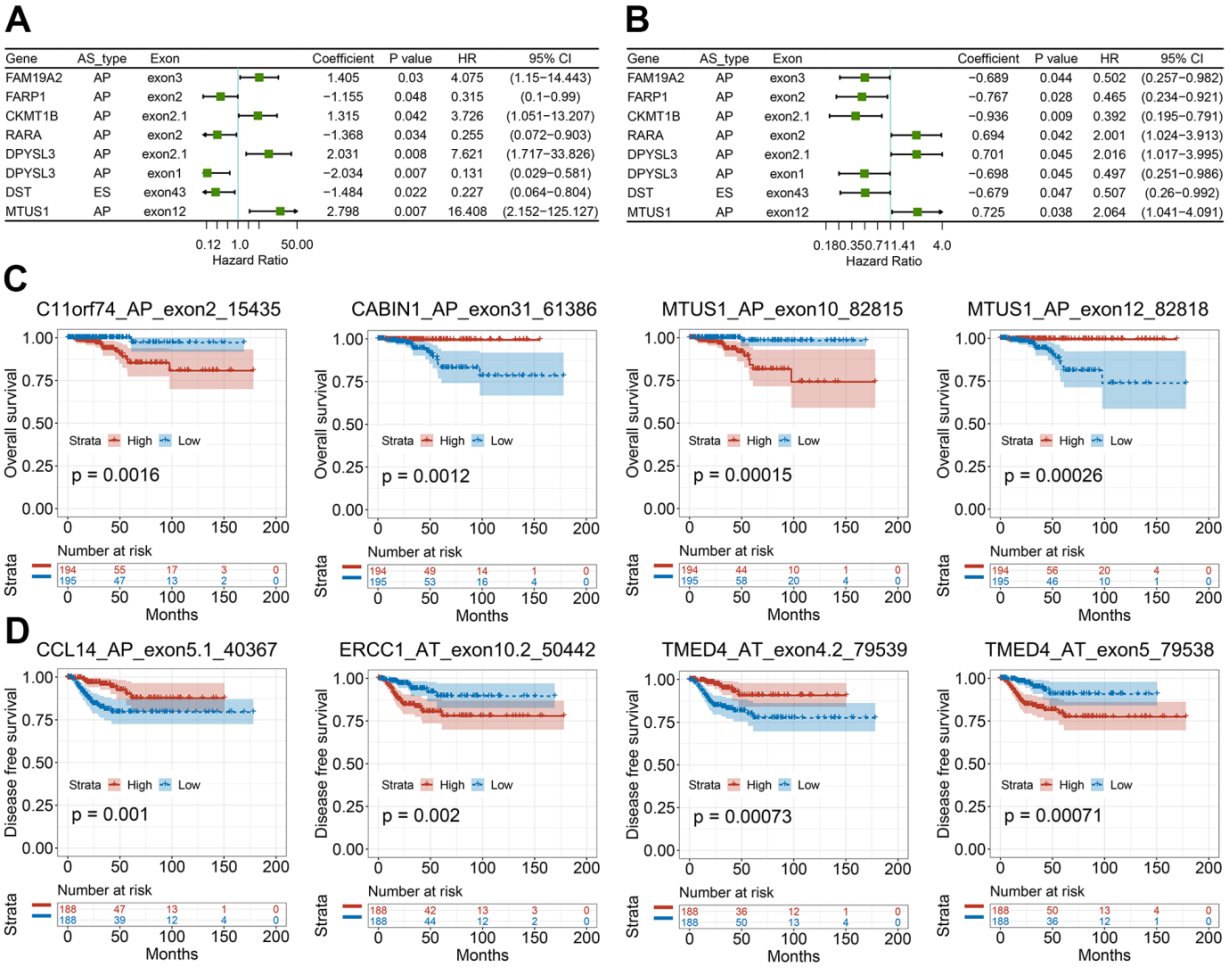
**Survival-associated CASEs in PTC.** (**A**, **B**) Forest plots of hazard ratios for eight CASEs simultaneously associated with OS (**A**) and DFS (**B**). (**C**, **D**) Kaplan-Meier curves of representative genes associated with OS (**C**) and DFS (**D**).

We then studied the correlation between our recognized CASEs and the PTC patients’ prognosis., PTC patients were separated into two groups on the basis of the median PSI value as per CASE. Based on the univariate survival analysis results, 90 AS events were remarkably linked with OS, and 113 AS events were significantly associated with DFS. Among these CASEs associated with survival, eight CASEs were linked with OS and DFS at the same time (Figure 6A, 6B). As presented in Figure 6C, 6D, the patients could be stratified according to the PSI value to form an obvious Kaplan-Meier curve in the OS and DFS survival analyses. Taken together, the above-mentioned results straightway implied that CASEs are not only biologically significant, but are also involved in PTC development.

### PTC feature gene selection and prognosis model construction

**Figure 7:**
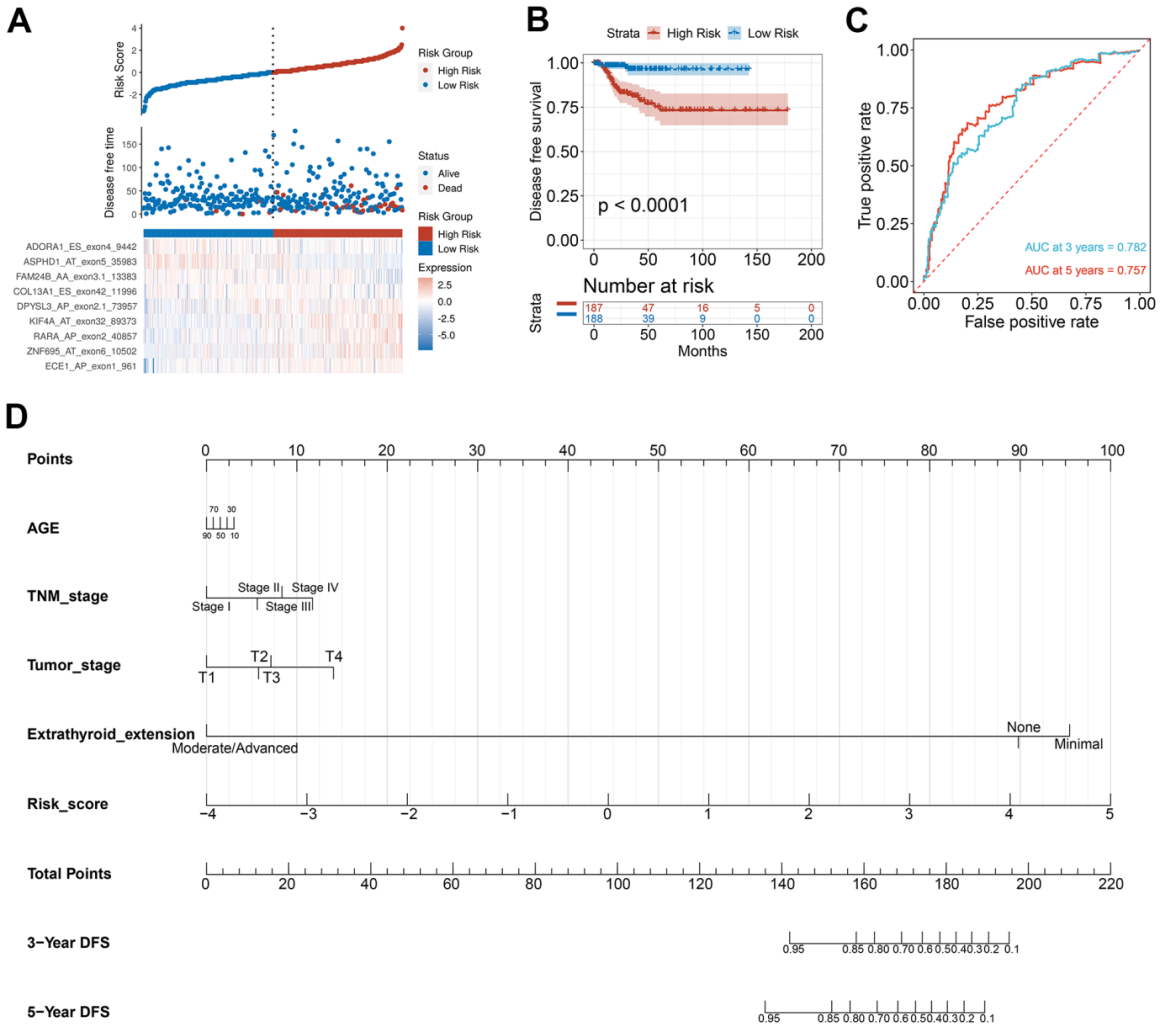
**Selection of PTC feature genes and construction of a prognosis model.** (**A**) Identification capability of the prognostic model of the low-risk and high-risk groups. (**B**) Kaplan-Meier curves of prognostic model for DFS of PTC patients. (**C**) ROC curves of prognostic model for the 3- and 5-year DFS of PTC patients. (**D**) Nomogram for predicting PTC patient’s 3- and 5-year DFS.

To verify the combined AS events’ predictive effect, the PTC feature genes were selected and a prognosis model was constructed. Using the LASSO Cox analysis following the univariate Cox analysis, nine CASEs with an upregulated parent gene were chosen to construct the ultimate prognostic model (Figure 7A). These nine gene signatures displayed good prognosis classification effects in the TCGA cohort (Figure 7B). The AUC curves of the 3- and 5-year DFS were 0.782 and 0.757, respectively, suggesting that these nine genes can function as prognostic markers of PTC (Figure 7C). To validate the prognostic association of identified CASEs in another independent cohort, we analyze our established prognostic model in HNSC and COAD. We observed the risk score associated with DFS in patients with HNSC and COAD, respectively (Supplementary Figure 2). These events indicate the prognostic association of our identified CASEs can be validate in HNSC and COAD, which mean our established prognostic model could be applied in other cancer types. We randomly divide the TCGA SpliceSeq data in PTC into test cohort (70%) and validate cohort (30%), and establish the nomogram with the test cohort (Figure 7D). Calibration curves confirm the accuracy of our nomogram for predicting 3- and 5-year DFS in both test and validate cohort (Supplementary Figure 3A). Decision curve analysis curves validate the value of clinical application of our nomogram in both test and validate cohort (Supplementary Figure 3B).

### AS-based clusters are remarkably linked with prognosis, molecular characteristics, and immune characteristics

**Figure 8:**
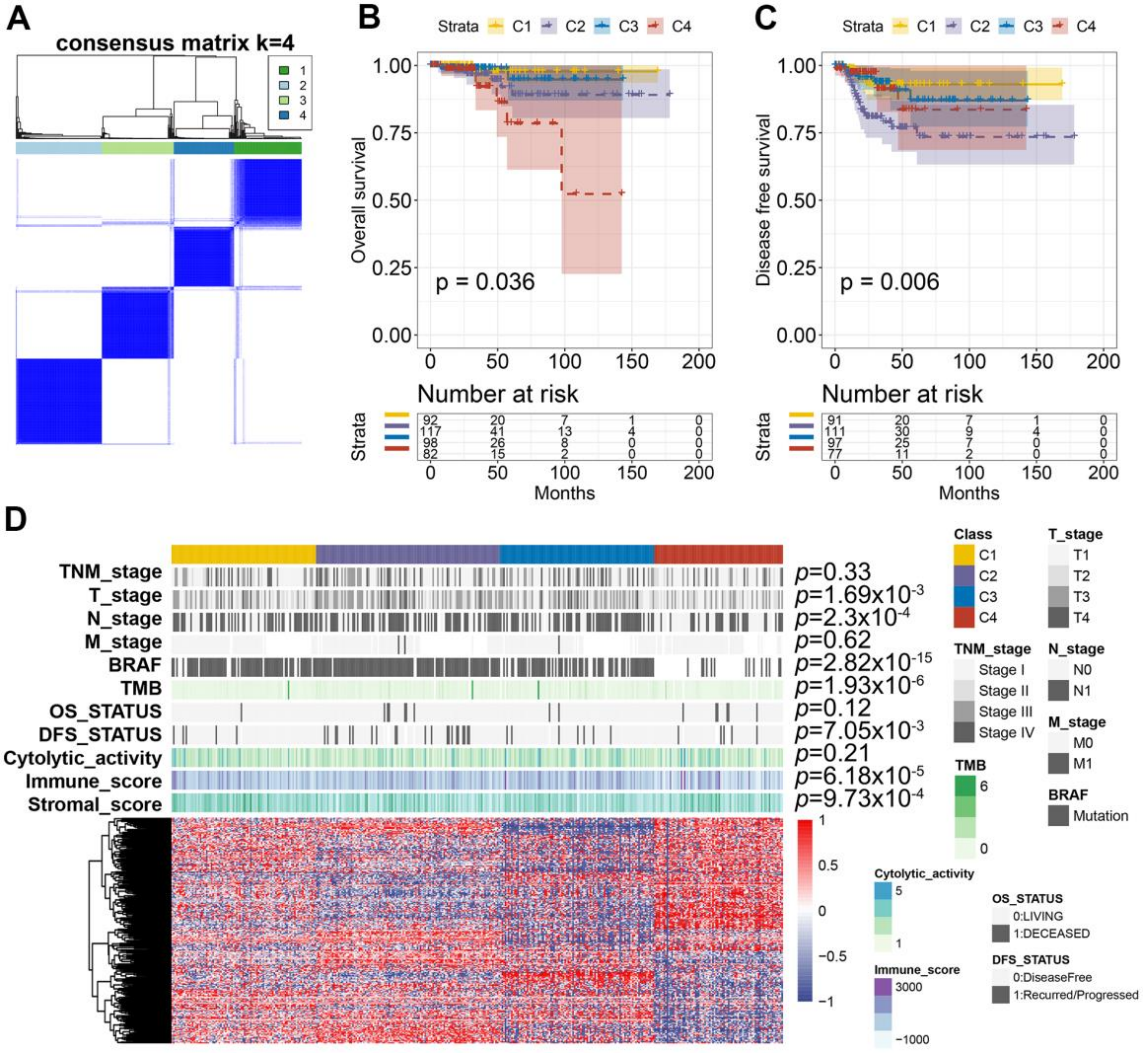
**AS-based clusters significantly associated with prognosis, molecular characteristics, and immune features.** (**A**) Consensus clustering analysis identified four clusters. The white (consensus value = 0, samples never clustered together) and blue (consensus value = 1, samples always clustered together) heatmap display sample consensus. (**B**) Kaplan-Meier curves show the OS for four AS-based clusters. (**C**) Kaplan-Meier curves show the DFS for four AS-based clusters. (**D**) Heatmap shows the molecular characteristics associated with clinical, molecular, and immune features among the four clusters.

To identify the distinct molecular subtypes correlated with prognosis and immune response, a consensus unsupervised analysis of all PTC tumor samples was performed. By adjusting the optimal parameters, four CAS-based molecular subtypes were identified (Figure 8A). Next, we explored the clinical values of the identified CASE-based clusters. The correlation between cluster and prognosis was evaluated using Kaplan-Meier analysis. Clusters were linked with different patterns of prognosis; cluster 1 was linked with favorable OS and DFS (Figure 8B, 8C). Cluster 4 was associated with shorter OS, while cluster 2 was associated with shorter DFS (Figure 8B, 8C). As shown in Figure 8D, the whole distribution of different molecular characteristics, such as TNM stage, BRAF, tumor mutation burden (TMB), and survival status (DFS and OS) in PTC samples between clusters, was significantly different. For instance, tumors classified as C4 had less BRAF mutants and lower TMB.

**Figure 9:**
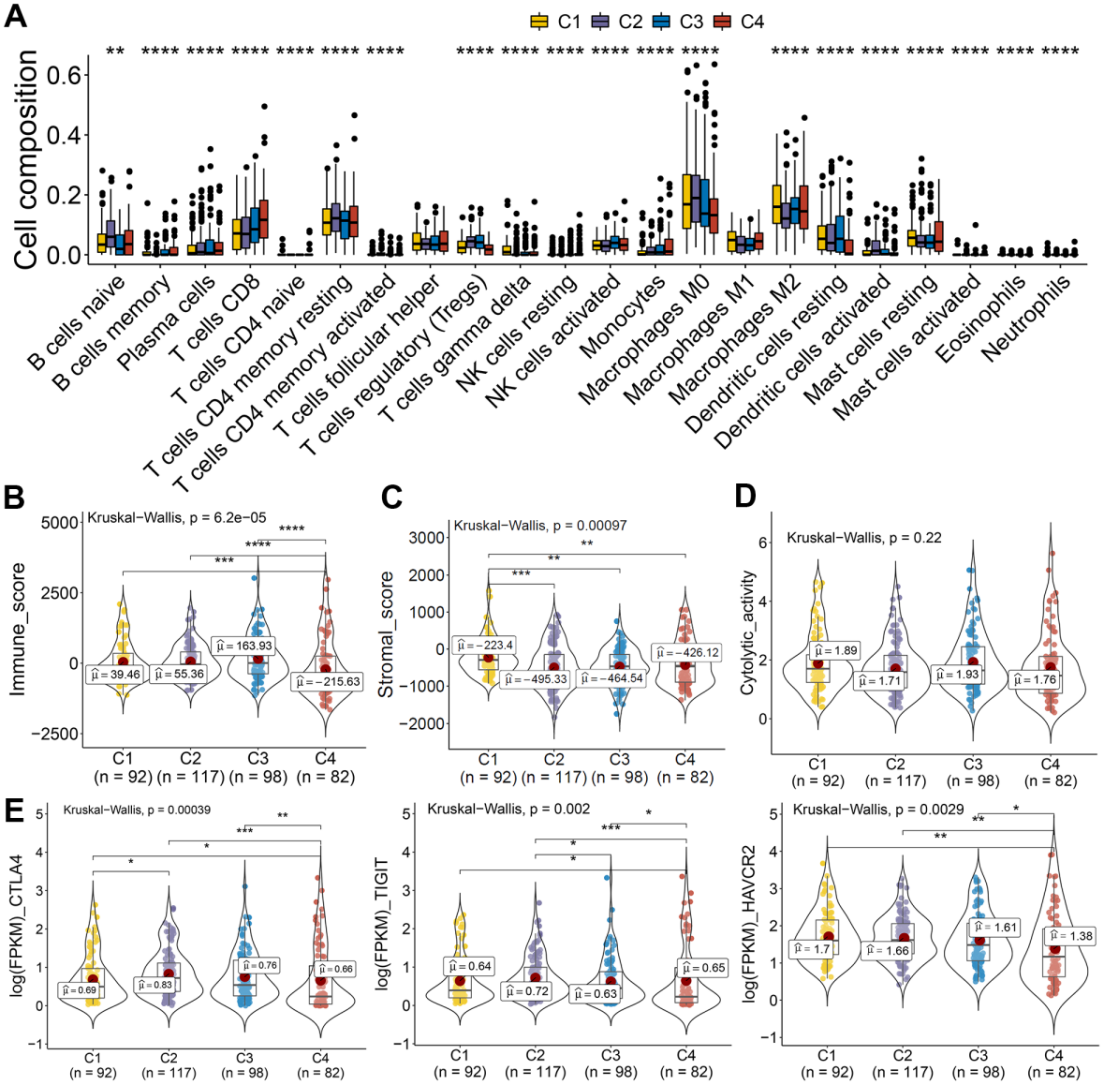
**AS-based clusters significantly associated with immune features.** (**A**) Bar plots of the relationship between AS-based clusters and infiltrate immune cell types. (**B**–**D**) Immune score, stromal score, and cytolytic activity between AS-based clusters. Data were analyzed using Kruskal-Wallis tests. (**E**) Expression level of immune inhibitory molecules among the four clusters.

The tumor microenvironment participates in cancer progression; the immune microenvironment differences among clusters based on CASE were investigated. Immune cell composition analysis revealed that C4 was linked with higher CD8 T cells, lower macrophage M0, and dendritic resting cells (Figure 9A). We computed the immune and stromal scores by the ESTIMATE algorithm, so as to quantify the stromal cell enrichment and immune cell infiltration in tumor tissues. Interestingly, cluster C4 was associated with low immune score, while C1 was correlated with higher stromal score (Figure 9B, 9C). Additionally, immune cytolytic activity analysis showed that C4 had lower cytolytic activity than that showed by other clusters (Figure 9D). We also observed lower expression of immune inhibitory molecules, like CTLA4, TIGIT, and HAVCR2, in cluster 4 than that in other clusters (Figure 9E). These results indicated the potential low immune response of cluster 4, which could explain the poor prognosis of cluster 4. Taken together, these results indicated that PTC showed different AS patterns. Clusters based on AS may function both as a prognostic indicator and as a promising index to find molecular targeted therapies and immunotherapeutic strategies for PTC.

## DISCUSSION

AS provides a mechanism for cells to diversify the proteome; however, AS also plays a critical part in the initiation or maintenance of malignancy [13, 32]. Exploration of AS patterns will broaden the understanding on the transcriptome molecular mechanisms in PTC. In this study, we integrated the AS event profiles and TCGA clinical information of patients with PTC, and identified PTC-related AS events and molecular signatures. The SF-AS network was also constructed to deepen our understanding of the regulatory mechanisms. We set up a prognostic model that successfully predicted the survival of PTC patients. Through CASE clustering, the PTC patients were stratified into subgroups with different survival results and immune features.

With the help of next-generation sequencing method, the analysis of the whole-genome sequence data and genome splicing data has been made possible. Recently, some studies have performed SpliceSeq analyses to form AS profiles and construct prognostic signatures for various cancers, including HNSC, colorectal cancer, non-small cell lung cancer, as well as pancreatic cancer [33–36]. However, the number of studies related to PTC is limited. We first identified the differentially expressed AS events between PTC tumors and adjacent healthy tissues. Results of functional enrichment analyses suggest that CASEs are enriched in pathways associated with tumor carcinogenesis, such as P53, epithelial mesenchymal transition, KRAS, IL6-JAK-STAT3, apoptosis, and MYC signaling. Our findings indicate that AS events play their biological roles in the tumor development. To explore the downstream mechanism of these CASEs, we performed a PPI network analysis. UBE2C and AURKB were recognized as hub genes. Notably, they both play crucial parts in tumor development, metastasis, and drug resistance [37–41]. For example, UBE2C can be increased by estrogen and accelerates epithelial-mesenchymal transition through p53 in endometrial cancer [41]. AURKB is a novel drug target for patients with non-small cell lung cancer with gained resistance to the therapy against EGFR [39]. These findings suggest the novel targets for PTC and pave the way for future clinical applications.

As the main upstream regulator of AS events, SF is crucial in cancer progression by mediating the AS process. A regulation network between SFs and CASEs was established and SFs CELF1 was observed as central in the regulatory network. CELF1 is in the RNA-binding protein family participating in multiple aspects concerning RNA processing (splicing and mRNA stability) [42]. CELF1 was involved in tumorigenesis and metastasis. By targeting ETS2 in colorectal cancer, it enhanced cell migration, invasion, as well as chemoresistance [43]. Wu et al. reported that siRNA-mediated knockdown of the CELF1 gene inhibited the multiplication of lung cancer cells [44]. Teng et al. profiled the transcriptomic signature in PTC which were linked with carcinogenesis and aggressiveness. Our study further broadens the understanding on the alterations in transcriptomes and the mechanisms underlying these alterations.

Considering the heterogeneity of malignant cells and the complexity of TME in PTC, the molecular markers of AS are still urgently needed. A risk score model was established and patients with different survival rates were effectively divided. A nomogram was also constructed to clearly predict the patients’ clinical prognosis. This study identified the CASE-based molecular subtypes to stratify the PTC patients’ prognosis. We identified four clusters with distinct prognoses and molecular characteristics. High levels of immune infiltration and immune inhibitory molecules were considered as biomarkers for better prognosis and reaction to immune checkpoint blockade therapy [45–47]. PTC samples classified as cluster 4 have lower immune and cytolytic activities. The immune inhibitory molecule expression, like PDCD1, CTLA4, and TIGIT, was lower in cluster 4 than in other clusters, implying that they may not respond to immune therapy. Cluster 4 has a shorter DFS than that of other clusters. These results imply that there is much variability in the property of the CASEs that have clinical significance.

Taken together, the current study established a global profile based on the differentially expressed AS events in patients with PTC, which is significant in decoding the AS events’ functional contribution in PTC. Our findings should promote continuous efforts to find new genomic molecules for clinical cancer treatment. Additionally, the further recognition of CASE-associated subtypes and establishment of the SF-AS network can provide new understanding to the in-depth investigation of splicing-related mechanisms.

## MATERIALS AND METHODS

### AS events curation process

The clinical data, gene expression, and corresponding mutation annotation files were downloaded from the databank of TCGA (https://portal.gdc.cancer.gov/) in thyroid cancer (THCA). The PSI values for THCA AS events were downloaded from the databank of TCGASpliceSeq (http://bioinformatics.mdanderson.org/TCGASpliceSeq/). Only patients with (1) PTC, (2) corresponding gene expression data, and (3) relatively complete clinical information on age, sex, TNM stage, and survival were retained. We utilized the ESTIMATE algorithm to compute the immune and stromal scores so as to count the percentage of stromal and immune cells in tumor samples [48]. The CIBERSORT method was used to characterize the composition of complex tumor tissues, including seven T-cell types, naïve and memory B cells, plasma cells, natural killer (NK) cells, and myeloid subsets [49]. The maftools (version 2.6.05) were used to assess the tumor mutational burden (TMB) of a tumor genome.

### Systematic presentation of AS events profiling

PSI values were processed by TCGA SpliceSeq, rating from zero to one, which was a typical, intuitive ratio to quantify splicing events. To obtain reliable AS events, strict filtering conditions were used (percent of samples with ≥75 PSI values, mean PSI value of ≥0.05). The UpSet plots drawn using the UpSetR (v1.4.0) package was utilized to show the interactive sets between different AS types [50]. Circos plots drawn using Circlize (v0.4.10) package were used to show the details of filtered AS events, CASEs, and their parental genes in the chromosomes [51].

### Cancer-associated AS event identification and functional enrichment analysis

The limma (v3.42.2) package was utilized to conduct a paired differential expression analysis of the PSI value of AS events between PTC and corresponding healthy tissues to identify CASEs [52]. AS events with |log_2_^FC^| of ≥0.1, adjusted P of <0.05 (adjusted by Benjamini and Hochberg (BH)) were selected as CASEs for further analysis. The clusterProfiler (v3.14.3) package was utilized to conduct Kyoto Encyclopedia of Genes and Genomes (KEGG) and Gene Ontology (GO) pathway function enrichment analysis on the CASE’s parent genes [53]. To identify the differences between PTC and paired normal tissues in pathways and biological functions, a gene set enrichment analysis (GSEA) was performed using the fgsea (v1.12.0) package. In addition, the Search Tool for the Retrieval of Interacting Genes/Proteins (STRING, v11.0) was applied to predict the protein-protein interaction (PPI) network between the CASE’s parent genes. The interaction network was visualized using Cytoscape (v 3.7.0) [54]. At the same time, cytoHubba and MCODE were utilized to recognize the module hub genes according to the STRING results in the Cytoscape.

### Build splicing correlation network

Data of 71 human RNA-binding splicing regulatory proteins were obtained from the SpliceAid-F database [31], which was retrieved by screening literature and databases manually. The relationship between the AS’s PSI value and SF’s expression was calculated to determine whether SF was significantly correlated with CASEs (|R| > 0.5, adjusted P < 0.01, adjusted by BH). We visualized the correlation network using Cytoscape (v 3.7.0) [54].

### Survival analysis

According to the PSI value of each CASE (median cut), patients with PTC were separated into two groups. HRs and the corresponding 95% CIs of various CASEs based on OS and DFS were calculated using univariate Cox regression analysis to search for differentially expressed and prognostic AS. Patients’ survival between the two groups was compared using Kaplan-Meier analysis. The differences were tested using the log-rank test.

### Construction of the risk score prognosis model

To explore the correlation between CASEs and DFS, some AS events were screened from the DFS-associated CASEs in the univariate regression analysis, in which parent genes were also significantly upregulated, and the least absolute shrinkage and selection operator (LASSO) and multivariate Cox regression analyses were conducted. The Glmnet R package (v4.0-2) was utilized to perform LASSO Cox regression analysis on candidate AS events. To assess the contribution of each AS event to DFS, a multivariate Cox regression analysis was performed. Finally, nine AS events were chosen to set up a risk score prognosis model. The predictive performance of this model was assessed through an ROC analysis which was time-dependent.

### Nomogram construction and validation

To predict the prognosis of PTC, the rms package (v6.1-0) was used to generate a nomogram according to the independent prognostic factors recognized by the multivariate analyses to explore the 3- and 5-year DFS of PTC patients.

### Assessment of the correlation with clinical and immunological characteristics

To make sure the PTC cohort was clustered in unbiasedly and unsupervised, the R package Consensus Cluster Plus (v1.50.0) was used to perform hierarchical consensus clustering (utilizing Kmdist and Pearson correlation coefficient distance) [55]. We made an analysis on the correlations between clusters, clinical pathological variables (TNM stage, T stage, N stage, and M stage), survival status (OS and DFS), molecular alteration (BRAF mutation), and immune characteristics (stromal score, immune score, immune cytolytic activity, and tumor mutational burden).

### Statistical analysis

All statistical analyses were conducted using the R software (v3.6.1), with a significance level set of 0.05 (unless otherwise stated). We used Pearson’s R to compute the correlation between the two continuous variables. The data with normal distribution were compared via analysis of variance test and student’s t-test; Wilcoxon signed-rank test and Kruskal-Wallis tests were applied to analyze data with abnormal distribution (*P ≤ 0.05, ** P ≤ 0.01, *** P ≤ 0.001, or **** P ≤ 0.0001). To make a comparison between categorical variables, Fisher’s exact test and Pearson’s chi-square test were utilized.

## Supplementary Material

Supplementary Figures
