# Supplementary material for: Comprehensive analysis of aberrant alternative splicing related to carcinogenesis and prognosis of papillary thyroid cancer
Source: Aging (Albany NY). 2021 Oct 8;13(19):23149–68. doi: 10.18632/aging.203608 (PMC8544310; doi:10.18632/aging.203608)
Supplement: Supplementary Figures [file aging-13-203608-s001.pdf]

SUPPLEMENTARY FIGURES

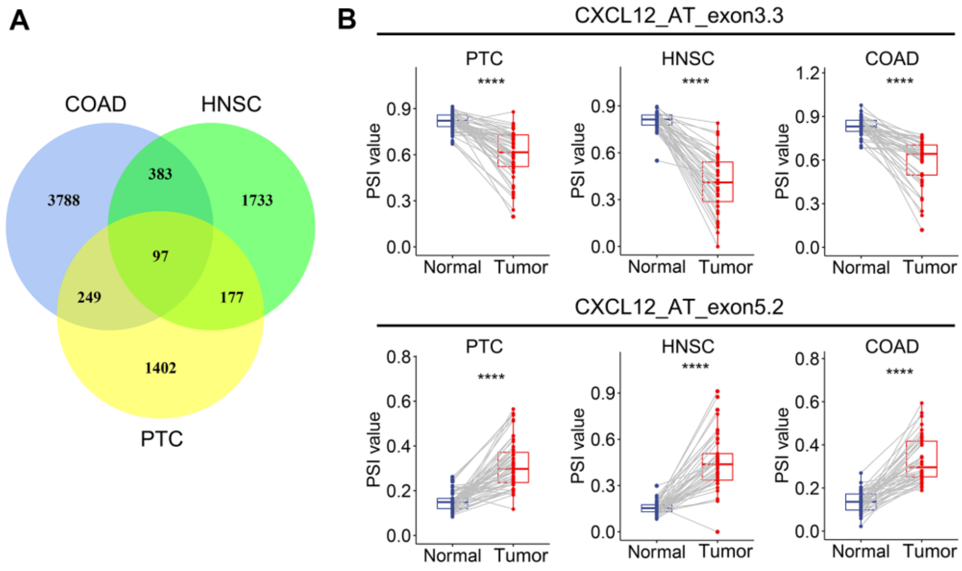

Supplementary Figure 1. Shared CASEs in PTC, HNSC and COAD. (**A**) Venn gram of CASEs in PTC, HNSC and COAD. (**B**) Representative common CASEs in PTC, HNSC and COAD. AS events with $|log_2^{FC}|$ of ≥0.1, adjusted P of <0.05 (adjusted by Benjamini and Hochberg (BH)) were selected as CASEs. ****, *P* < 0.0001. CASEs, cancer-associated AS events; AS, alternative splicing; PTC, papillary thyroid cancer; HNSC, head and neck squamous cell carcinoma; COAD, colon adenocarcinoma.

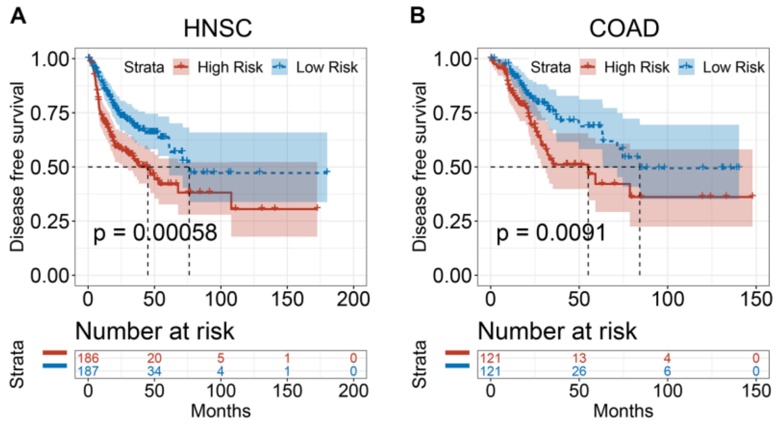

Supplementary Figure 2. Validation the established prognostic model in HNSC and COAD. (**A**) Kaplan-Meier curves of prognostic model for DFS of HNSC patients. (**B**) Kaplan-Meier curves of prognostic model for DFS of COAD patients. DFS, disease free survival; HNSC, head and neck squamous cell carcinoma; COAD, colon adenocarcinoma.

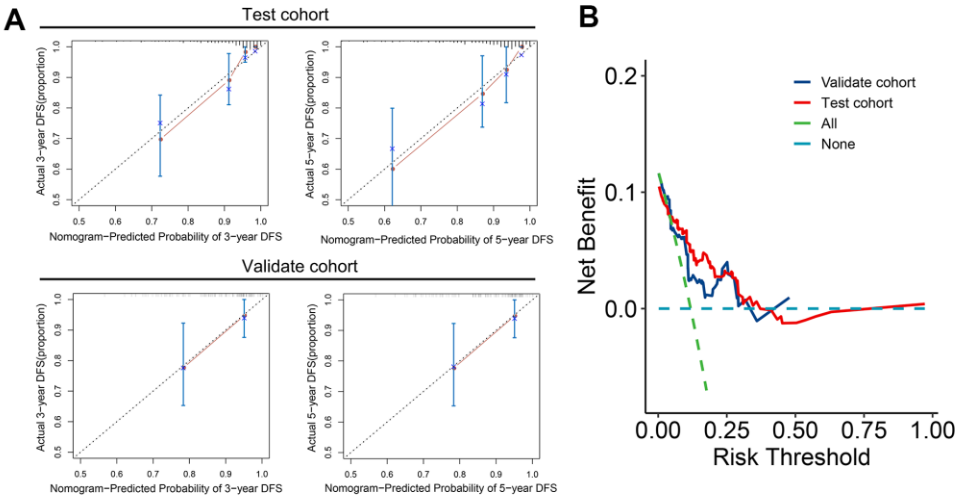

Supplementary Figure 3. Validation the accuracy and clinical application of established nomogram. (**A**) Calibration curves confirm the accuracy of our nomogram for predicting 3- and 5-year DFS in both test and validate cohort. (**B**) Decision curve analysis curves validate the value of clinical application of our nomogram in both test and validate cohort.
